# Prediction of drug concentrations in humans for long-acting injectable suspensions by a semi-mechanical muscle compartment model: a case study of paliperidone palmitate

**DOI:** 10.3389/fphar.2025.1507828

**Published:** 2025-07-09

**Authors:** Panpan Yu, Mengjun Zhang, Xiong Jin, Keheng Wu, Sihui Long, Shishi Cheng, Long Fu, Xiao Xu, Jie Liu, Dan Liu, Xue Li, Bo Liu, Jian Xu

**Affiliations:** ^1^ Livzon Pharmaceutical Group Inc., Livzon Corporate, Zhuhai, Guangdong, China; ^2^ School of Chemical Engineering and Pharmacy, Wuhan Institute of Technology, Wuhan, China; ^3^ Yinghan Pharmaceutical Technology (Shanghai) Co., Ltd., Shanghai, China

**Keywords:** paliperidone, semi-mechanical muscle compartment model, long-acting injectable, nanocrystals, prediction in human subjects

## Abstract

Long-acting injectable formulations, such as paliperidone palmitate extended-release injectable suspension, have been designed to release medicines slowly and sustainably. Developing models that simulate drug release from long-acting injectable formulations *in vivo* is challenging. A novel approach to modeling and simulating complex and multiphasic drug pharmacokinetics (PK) is provided in this article to facilitate development of long-acting formulations. By segmenting nanocrystalline particles according to their different sizes, the absorption delays of each segment were obtained from the results of the PK study in dogs. In addition to the lag time for each segment, all other parameters, including physicochemical parameters such as drug solubility, density and diffusion coefficient, as well as pharmacokinetic parameters related to clearance, elimination and distribution, were introduced into the model to establish a muscle compartment model for use in humans. By using this model, the injectable suspensions paliperidone samples were predicted to have a long release of 90–100 days *in vivo*.

## 1 Introduction

Nanocrystals, also known as nanosuspensions in the liquid formulations, are nano- or micro-scale dispersions formed by dispersing poorly soluble drug particles in water or oil with a small amount of surfactant or polymer as a stabilizer. Nanocrystals are engineered to enhance saturation solubility, increase dissolution rate, improve chemical stability, and reduce toxicity of formulations. They have shown promise in developing controlled or extended-release formulations that facilitate efficient disease management. Their controlled release is influenced by factors such as particle size distribution, particle stability, coating materials or suspending agents, and storage in tissues (Kalhapure et al., 2022).

When a nanocrystalline drug is injected into muscle tissue, the drug particles are initially released from the formulation. The characteristics of the formulation influence its release rate. Following the initial release, the nanocrystals dissolve in the muscle’s interstitial fluid. The dissolution and absorption rates of these nanocrystalline particles are affected by their size (Sun et al., 2012; Jarvis et al., 2019), due to surface area and governed by solubility principles. In particular, smaller nanocrystals have a higher surface area-to-volume ratio and more surface contact with biological fluids, resulting in faster dissolution rates. This results in faster absorption of the drug into the systemic circulation. Additionally, the overall behaviour of nanocrystal drug formulations is also influenced by factors such as the physicochemical properties of the drug, the interactions between the drug particles and muscle tissue, blood flow within the muscle, and local metabolism of the drug (Jarvis et al., 2019).

Despite growing interest in long-acting injectable products for the treatment of chronic diseases, the complexity and multiphase drug release process pose significant challenges in accurately modelling their pharmacokinetic properties (Gomeni and Bressolle-Gomeni, 2021). To facilitate drug development, knowledge on how drugs are distributed in the body and how drugs behave in different species is essential for pre-clinical testing in humans. Mathematical models are developed and used to predict drug concentrations in the human body.

Physiologically based pharmacokinetic (PBPK) models are extensively used across various disease domains to predict the PK of new drugs, simulate different clinical scenarios, and improve existing treatments (Zhang et al., 2021a; Li X. et al., 2022; Zhang et al., 2021b; Li J. et al., 2022; Zhang et al., 2023; Wu et al., 2024). These models also assess how changes in drug formulation might affect PK (Rajoli et al., 2015). To identify optimal dosage and predict long-acting release rates for the design of long-acting formulations, various modeling approaches have been applied (Gomeni and Bressolle-Gomeni, 2021; Rajoli et al., 2015).

In this study, we developed a muscle compartment model to simulate the concentration of a drug in the body after injection of long-acting nanocrystals in humans. Long-acting injectable forms of paliperidone palmitate, an antipsychotic drug for schizophrenia and schizoaffective disorders, were selected for model construction. Paliperidone palmitate is typically formulated as a nanocrystal suspension. They exhibit extremely low solubility and slow dissolution rate and paliperidone palmitate undergoes hydrolysis into paliperidone once it enters the systemic circulation. This slow release helps maintain consistent medication levels in the bloodstream, which can enhance treatment adherence and better relieve symptoms (Owen, 2010; Citrome, 2010; Bishara, 2010; Gilday et al., 2012; Hoy et al., 2010; Carter, 2012; Chue and Chue, 2012). Paliperidone primarily binds to ɑ_1_ – acid glycoprotein and albumin (Chue and Chue, 2012), with a plasma protein binding rate of 74% at concentrations ranging from 50 to 250 ng/mL (Chue and Chue, 2012). It undergoes minimal hepatic metabolism and is eliminated through various metabolic pathways, each contributing up to 6.5% to the biotransformation of the total dosage (Vermeir et al., 2008). For this study, long-acting injectables were designed and references such as Invega Sustenna^®^, was selected.

Nanocrystalline particles were subdivided according to their sizes, and the absorption delay time of each group of particles was derived from the results of PK study in dogs. Combined with other physicochemical parameters as well as PK parameters, a muscle compartment model was developed in humans. The model can predict the release profile of paliperidone injectable suspensions for 90–100 days *in vivo*. In the future, this model will be used to study more cases to improve it predicting power and help its generalization. With further improvement and eventual perfection of this model in due time, it shall find wider applications.

## 2 Materials and methods

### 2.1 Modelling process

**FIGURE 1:**
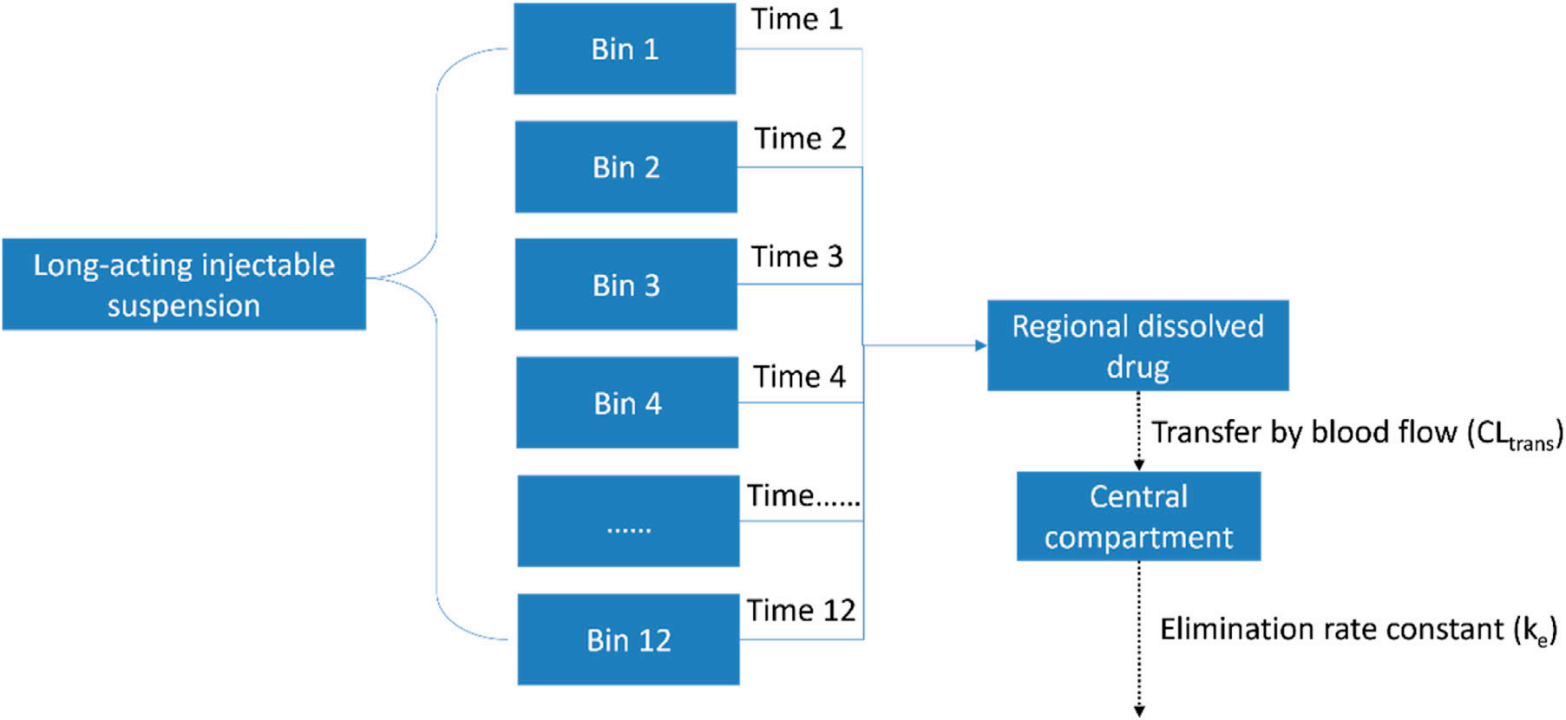
Modelling scheme of the semi-mechanical muscle compartment model.

Depending on particle size, the model used twelve bins to represent different dissolution times (Time-1 to Time-12) and different ‘lag times’ – the delay from the injection of nanocrystalline particles to their entry into the systemic circulation. After the drug was dissolved in the injection area, it was transferred to the central compartment. This transfer process is also considered as the drug being cleared and entering the circulatory system. More details of the modelling scheme are provided in Figure 1.

**FIGURE 2:**
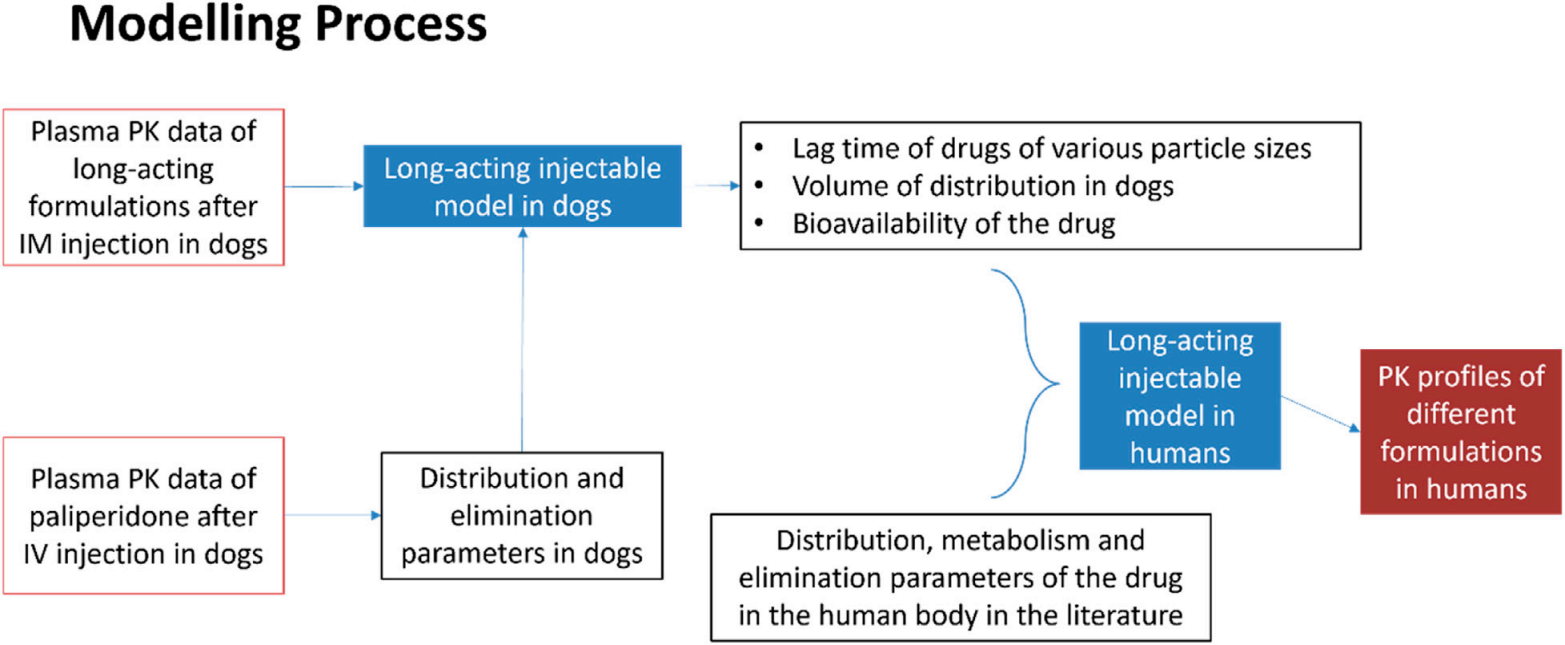
Modelling process of long-acting injectable model in humans.

In the modelling process, pharmacokinetic data such as distribution and elimination parameters for long-acting formulations following intramuscular (IM) injection in dogs, were initially used to develop a long-acting injectable model in dogs. The distribution and elimination parameters were obtained from pharmacokinetic study following intravenous injection in dogs. Using this model, other parameters such as lag times for various particle sizes, volume of distribution, and bioavailability of the drug formulation were subsequently derived. Then, together with parameters such as distribution, metabolism and elimination relevant to humans, all parameters were adjusted to establish the long-acting injectable model in humans. After validation, this model was used to predict the pharmacokinetic profile of long-acting formulations in humans. The details of the modelling process are shown in Figure 2.

### 2.2 Materials

The paliperidone palmitate extended-release injectable suspensions were designed and prepared by Livzon Pharmaceutical Group Inc. Reference suspension Invega Sustenna^®^ was purchased from Janssen Pharmaceutica N.V.

### 2.3 Measurement of drug’s solubility

The solubility of paliperidone was measured using the Shake-Flask Method and combined with computer simulation predicted values using the ALOGPS program (an online calculation tool that can predict the solubility of compounds.), and used as a theoretical reference. The Shake-Flask Method is a simple *in vitro* technique routinely used to assess equilibrium solubility (pH-dependent) or release kinetics. The method for assessing drug solubility requires consideration of composition of aqueous buffer, stirring time, sedimentation time, temperature, amount of solid excess, and phase separation methods. Still discrepancy exists between solubility determined *in vitro* and *in vivo* solubility due to the differences between *in vitro* and *in vivo* environments (e.g., the stirring rate of the Shake-Flask method is faster than the blood flow rate of muscle tissue, and the porosity of the dialysis membrane is inconsistent with capillaries.). The results should be interpreted with caution. Yet, the Shake-Flask Method is still suitable for rapid screening of compounds in early drug discovery stage and lead optimization.

Shake-Flask Method: Buffer solutions at various pH levels were prepared and added into a 100 mL measuring flask. Excess drug was weighed and added to the flask. The flask was shaken vigorously for 30s every 5 min, until the sample was no longer soluble. The flask was placed on an orbital shaker and shaken at 37°C. Ten millilitres of the sample was taken at 1 h, 2 h using a 0.2 μm filter to remove undissolved particles. The filtered solution was analysed using High-Performance Liquid Chromatography (HPLC). The solubility of the drug in mg/mL was calculated based on the concentration found in the solution.

### 2.4 Measurement of the size of the nanocrystals

The size of nanocrystals was measured using the Malvern Mastersizer 3,000. Water was used to dilute the paliperidone palmitate samples to a volume of 250 mL. Once well dispersed, the samples were analysed using parameter settings of refractive index of 1.56 and absorption of 0.01. The duration of the measurement was 30 s. The Mastersizer 3,000 software was used to analyse the diffraction pattern and to calculate the particle size distribution.

### 2.5 Animal study

#### 2.5.1 Pharmacokinetic studies in dogs

Beagle dogs (male, aged 7–9 months, weighing 9–11 kg) were supplied by Shanghai Jiagan Biotechnology Limited Company (China) for the pharmacokinetic studies. All experimental procedures adhered to the company’s ethics and regulations for animal experiments stated in regulatory sections. A 50 mg dosage of paliperidone palmitate was injected into the muscle tissue of a dog’s buttock by IM injection. T1 and T2 were paliperidone samples, and R, the reference sample, was the paliperidone palmitate injectable from Invega Sustenna^®^.

#### 2.5.2 Determination of drug concentration

##### 2.5.2.1 Plasma sample collection and processing

1) Sampling procedure After the injection was completed, timing began at 0 h before administration, 6 h after administration, 1 day, 2 days, 3 days, 4 days, 5 days, 6 days, 7 days, 8 days, 9 days, 10 days, 11 days, 12 days, 14 days, 16 days, 18 days, 20 days, 24 days, 28 days, 32 days, and 36 days (a total of 22 blood collection points). One milliliter blood sample was taken from the dog’s left forelimb vein and placed in a tube containing anticoagulant EDTA-K2. Immediately after blood collection, the blood collection tube was gently and completely inverted 3 times to mix with the anticoagulant.
2) Plasma separation The process of whole blood collection until separation of plasma was operated under ice bath conditions within 1 hour. The sample was placed in a 4°C cryogenic centrifuge and centrifuged at 3,500 rpm for 10 min to separate the plasma.
3) Sample storage The supernatant containing paliperidone was collected and stored at −70°C.

##### 2.5.2.2 Quantitative analysis of biological samples

For analysis, Liquid Chromatography (LC)/Mass Spectrometry (MS)/MS was used to measure the drug concentration in the plasma samples. The linear range of the concentration was from 0.500 to 500 ng/mL.

The chromatographic analysis was performed on a Shimadzu liquid chromatography system (Shimadzu, Japan), consisting an infusion pump, an auto-sampling system, a controller, a column temperature chamber and a degasser. The analytes were separated on an Eclipse Plus C_18_ column (4.6*100 mm, 3.5 μm, Agilent), and the mobile phase was a mixture of acetonitrile and 10 mM ammonium acetate (containing 0.1% formic acid) with a volume ratio of 45:55 for isocratic elution, with a running time of 2.5 min and a flow rate of 0.7 mL/min. The chromatographic conditions were as follows: column temperature at 40°C, auto-sampling system temperature at 4°C, injection volume of 0.5 μL. The needle wash solution and the needle wash mode were as follows: for R0, R1, R2 methanol-water (1:1, v/v) was used; for R3 methanol-acetonitrile-isopropanol-water (1:1:1:1, v/v) was used; and the needle wash procedure was performed in the external mode, with port rinsing before and after aspiration, followed by pump flushing after analysis.

Mass spectrometry was performed using a Triple Quad 5,500 triple quadrupole tandem mass spectrometer equipped with an Electrospray Ionizer (ESI) (AB Sciex, United States). The ion pairs used for quantitative analysis were as follows: Paliperidone m/z 427.2→207.2, and Internal Standard m/z 431.3→211.2. The mass spectrometry parameters were set as follows: the ion source gas was N_2_, the ion source gas 1 was at 50 psi, the ion source gas 2 was at 50 psi, and the curtain gas was at 40 psi; ion source temperature at 500°C, source injection voltage at 5500 V; Declustering Potential (DP) at 130 V, Collision Cell Exit Potential (CXP) at 13 V; collision energy for Paliperidone at 34 V, for internal standard at 39 V; positive ion mode and Multi Reaction Monitoring (MRM) scanning were used, with Unit resolution for Q1 and Q3, Collision Activated Dissociation (CAD) at 8 psi, Entrance Potential (EP) at 10 V, and a scan time of 40 ms.

##### 2.5.2.3 Data processing and analysis

Plasma drug concentration-time data and curves for individual subject animals and the mean, standard deviation, and curves for each formulation dosing group are given separately.

The non-compartmental model of Phoenix WinNonlin 8.3 software was used to calculate the pharmacokinetic parameters of the drug in Beagle dogs after administration.

Peak concentration (C_max_): measured value was used.

Time to peak (T_max_): measured value was used.

Area under the concentration-time curve (AUC_0-t_): calculated by the trapezoidal method.

${\text{AUC}}_{0-}\infty$ = AUC_0-t_ + C_t_/k_e_, C_t_ is the plasma drug concentration at the last measurable time point, k_e_ is the elimination rate constant.

Elimination half-life (t_1/2_) = 0.693/k_e_; Mean retention time (MRT) = AUMC/AUC.

### 2.6 Modelling work

#### 2.6.1 Model construction

When nanocrystals are injected into muscle tissues, their dissolution and absorption rates depend largely on their size. In the current model, all injected nanocrystals were grouped into N groups, each representing a different size range. Each group had a different delay before the drug began to be absorbed. The rate at which the drug dissolves was determined by the Diffusion Layer Model (Wang and Flanagan, 1999). As shown in Equations 1, 2, for nanocrystals of various sizes with corresponding absorption delay times, the mass change for each group of solids (Solid_n_), i.e., dissolution rate, was calculated.

$$\frac{d{Solid}_{n}}{dt}=0\;\left(if\;t\ll t{lag}_{n}\right)\text{or}=-N_{n}*\;\frac{D}{h}*4\pi *\;\sqrt[{3}]{\frac{3*\;{Solid}_{n}}{4\pi \rho }}*\left(\sqrt[{3}]{\frac{3*{Solid}_{n}}{4\pi \rho }}+h\right)*\left(S-\frac{Regional_{Dissolved}}{V_{region}}\right)\;\left(if\;t>t{lag}_{n}\right)$$ (1)

tlag_n_: time point after which the solid mass of the n^th^ bin begins to dissolve; N_n_: number of particles in the n^th^ bin; D: diffusion coefficient of paliperidone; h: thickness of diffusion layer; ρ: density of solid particles; S: solubility of paliperidone in the regional environment after administration; Regional_Dissolved_; dissolved mass of paliperidone in the regional environment V_region_: volume of the regional environment.

Equation 2 represents the rate of change in the mass of the locally dissolved drug (Regional_Dissolved_):

$$\frac{{d\;Regional}_{Dissolved}}{dt}=-\sum_{n}\frac{d{Solid}_{n}}{dt}-\frac{Regional_{Dissolved}}{V_{region}}*\;{CL}_{perf}$$ (2)

CL_perf_: clearance when the regionally dissolved drug being carried away by blood flow.

When the drug was dissolved and transferred to the central compartment, it underwent distribution and elimination as described in Figure 1. The change in the mass of the drug within the central compartment was calculated using Equation 3. The plasma concentration of the drug was then determined by dividing the mass of the drug (Mc) by the volume of the central compartment.

$$\frac{dMc}{dt}=\frac{{Regional}_{Dissolved}}{V_{region}}*\;{CL}_{perf}-Mc*ke$$ (3)

k_e_: elimination rate constant.

The model incorporated the drug’s physicochemical properties and pharmacokinetic parameters as inputs to capture the drug’s penetration, absorption, distribution, and elimination process by analysing the drug’s behaviour *in vivo* after IM injection in dogs. Through this analysis, the injected nanocrystals were divided into twelve bins (n = 12), each with a different size (range) and corresponding dissolution lag time, which best matched the pharmacokinetic data observed in dogs. This configuration indicated optimal modelling of drug dissolution kinetics.

**FIGURE 3:**
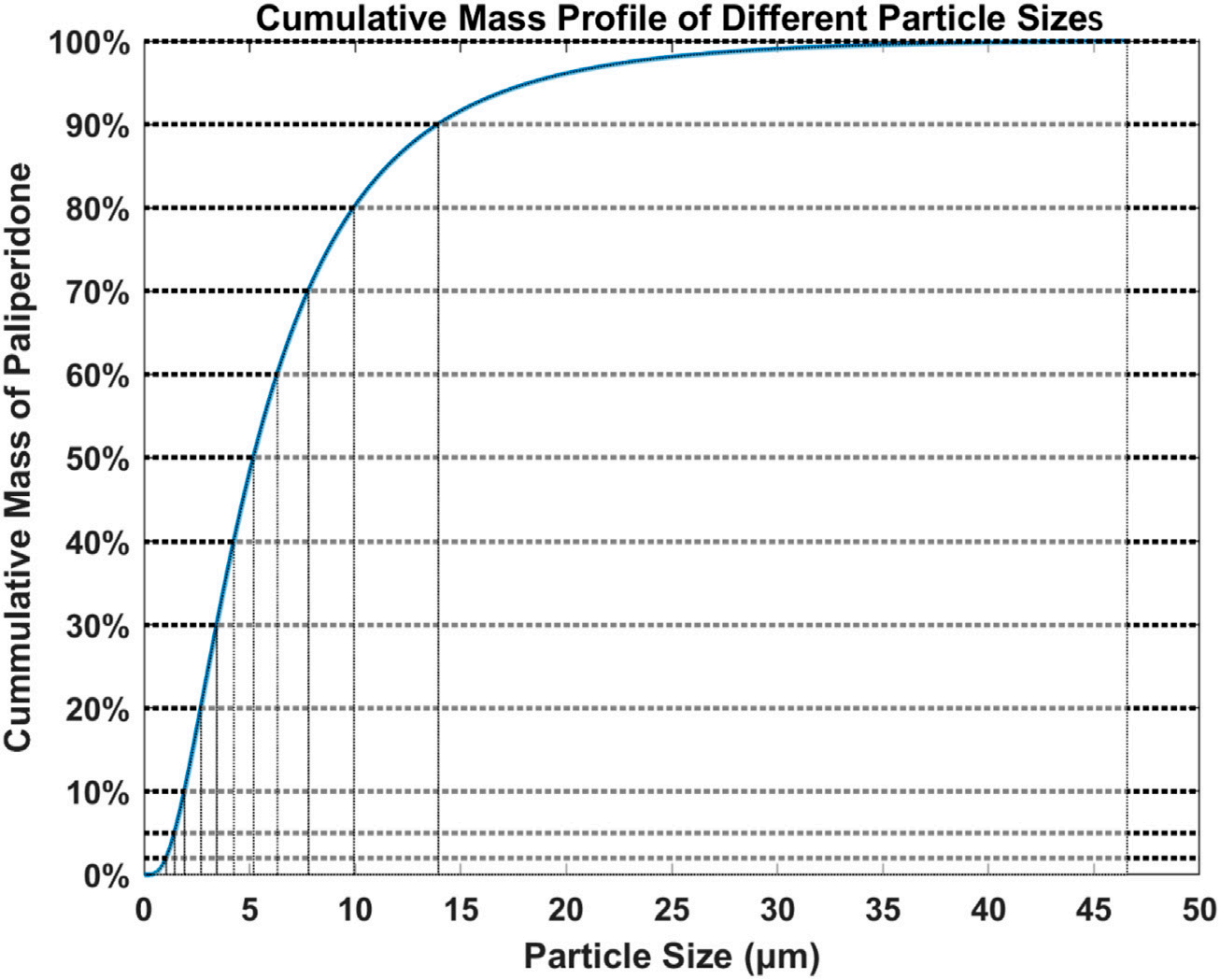
Cumulative mass profile of reference sample R with different particle sizes.

Based on the data collected from the particle size analysis, the cumulative mass percentage for each particle size was calculated and the particle size distribution was plotted as shown in Figure 3 (Reference sample R). The particles were divided into 0%–2%, 2%–5%, 5%–10%, 10%–20%, 20%–30%, 30%–40%, 40%–50%, 50%–60%, 60%–70%, 70%–80%, 80%–90%, and 90%–100% according to the total mass and were used as 12 bins. The average particle size in each bin was calculated.

#### 2.6.2 Simulation design and modelling software

Three hundred virtual individuals received a single dose of paliperidone palmitate with the same dosage as in the clinical trial study for the reference sample. The model employed MATLAB version R2018b using the source code and architecture of the PBPK model on B^2^O simulator (Version 3.0, Yinghan Pharmaceutical Technology (Shanghai) Co., Ltd.) to predict drug exposure. At CI% (Confidence Interval) upper and lower limits of 5% and 95%, respectively, the geometric means of all C_max_ and AUC were calculated and compared to observations. A difference of more than 2-fold change was considered significant.

## 3 Results

### 3.1 Parameters of paliperidone used to establish the long-acting injectable model in dogs

#### 3.1.1 Physicochemical parameters

The physicochemical parameters of paliperidone are shown in Table 1. The solid density (ρ) of paliperidone was obtained from the ChemSpider website (https://www.chemspider.com/Chemical-Structure.8028457.html). A 50 mg dosage was used to simulate the extended-release of paliperidone from an injectable suspension designed for prolonged release. Assuming 50 mg of paliperidone was provided in 0.5 mL, the depot volume was 0.5 mL. The diffusion coefficient (D) was initially calculated using the Stokes-Einstein Gierer-Wirtz estimation method (Evans et al., 2018) to be 5.34 × 10^−10^ m^2^/s in water at 37°C and fitted to the observed PK data to be 1.17 × 10^−11^ m^2^/s.

**TABLE 1:** Physicochemical and pharmacokinetic parameters of paliperidone in dogs.

Parameters	Symbol	Value
Solid density	ρ	1.2 g/cm^3^
Solubility	Cs	0.00704 mg/mL
Diffusion coefficient	D	1.17 × 10^−11^ m^2^/s
Dosage	—	50 mg
Clearance of paliperidone distributed in blood	CL_dist_	35 L/h
Diffusion layer thickness	h	3 μm
Half-life	t_1/2_	7.6 h
Elimination rate constant	K_e,dog_	0.0693 h^−1^
Volume of distribution	Vd_dog_	8.5 L
Bioavailability	F	1

#### 3.1.2 Pharmacokinetic parameters related to paliperidone injectable in dogs

Based on the pharmacokinetic results of the dog study, the clearance of paliperidone from the blood was fitted as CL_dis_ = 35 L/h and the diffusion layer thickness was 3 μm. The drug’s half-life in dogs was obtained from the published literature and ranged from 5.8 to 9.4 h (Janssen, 2006), with a determined mean of 7.6 h. The elimination rate constant K_e,dog_ was determined to be 0.0693 h^−1^ using the half-life of the drug. The volume of distribution (Vd_dog_) was calculated as 8.5 L, and the bioavailability was determined to be 100% from the pharmacokinetic analyses in the dogs. The pharmacokinetic parameters in dogs are shown in Table 1. In addition, the lag time for each bin of the reference sample (R) was also derived from the pharmacokinetic results of the dog study, which are detailed in Table 2.

**TABLE 2:** Lag time in hours for 12 bins.

Batch name	Bin 1	Bin 2	Bin 3	Bin 4	Bin 5	Bin 6	Bin 7	Bin 8	Bin 9	Bin 10	Bin 11	Bin 12
R	4.8	21.6	33.6	52.8	79.2	84	84	72	72	72	72	72
T1	1.2	4.8	12	19.2	28.8	60	72	72	72	72	144	144
T2	4.8	19.2	28.8	48	64.8	84	96	96	120	120	120	120

### 3.2 Establishment of the long-acting injectable model in dogs

**FIGURE 4:**
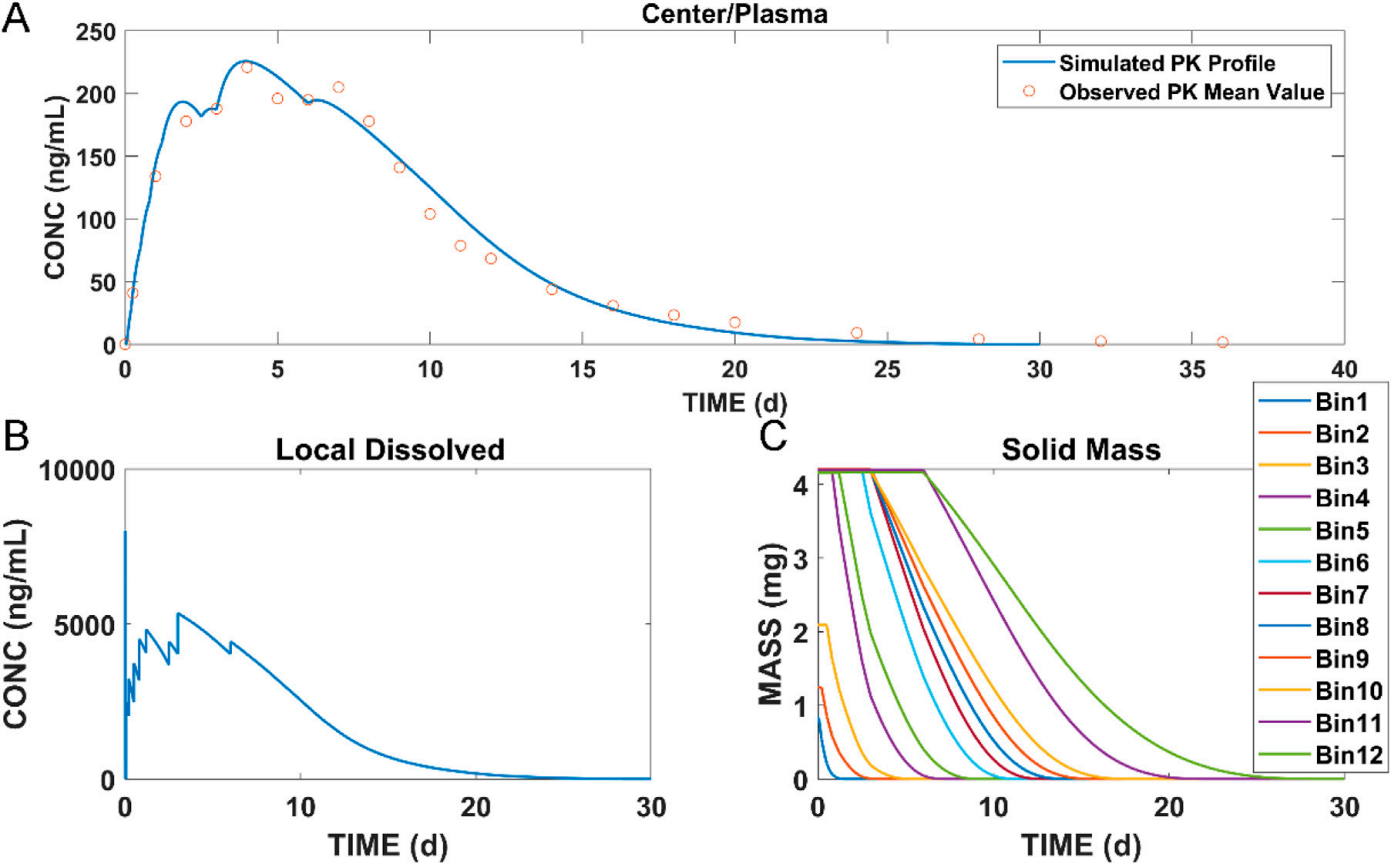
Simulation results of **(A)** plasma concentration of paliperidone in dogs; **(B)** concentration of dissolved paliperidone in local muscles; **(C)** local solid mass of undissolved paliperidone for each bin. (Continuous lines: simulated results; circles: observed mean value of concentration data at each time point).

The muscle compartment model for long-acting injectables in dogs was developed by combining data from pharmacokinetic studies in dogs and *in vitro* experiments. The plasma concentrations of paliperidone (continuous line) simulated from the model are shown in Figure 4A. Compared to the observations (circles), both the simulated results and the observations showed that paliperidone was cleared in the body around 35 days after injection. The simulated plasma concentration of paliperidone in dogs was in good agreement with the observations, and the prediction was less than 2-fold that of the observations. The calculated and observed results of AUC_0-t_ and C_max_ and their respective ratios are shown in Table 3.

**TABLE 3:** Comparison of simulated and observed results in dogs.

Batch	Simulated AUC_0-t_ (ng/mL*hr)	Observed AUC_0-t_ (ng/mL*hr)	Ratio^a^	Simulated C_max_ (ng/mL)	Observed C_max_ (ng/mL)	Ratio^b^
R	64,962	59,753	1.087	262.7	286.0	0.919
T1	53,963	53,546	1.008	225.8	221.0	1.022
T2	46,808	44,581	1.050	209.9	194.0	1.082

^a^ Ratio: the ratio of the simulated AUC_0-t_ to observed.

^b^ Ratio: the ratio of the simulated C_max_ to observed.

The model was also used to simulate the concentration of dissolved paliperidone in local muscles, and the results are shown in Figure 4B. The linear fluctuations in the curve were discontinuous due to the limited number of bins divided. As the number of bins increases, the curve should be closer to the actual situation, becoming continuous and smooth. Figure 4C shows the change in undissolved paliperidone over time for each bin of the simulation. Since large nanocrystals dissolved more slowly when injected into the muscle compared with smaller ones, the particles in bins 11 and 12 did not dissolve in the muscle until Day 6 and completely dissolved within 30 days.

### 3.3 Establishment and validation of long-acting injectable model in humans

After the model was developed in dogs, it was extrapolated for use in humans. The elimination rate constant (ke) was calculated using the clearance (CL) and volume of distribution (Vd) values from the study by Samtani et al. (2009). In detail, the clearance was reported as 4.95 L/h and the Vd as 391 L, leading to the calculation: Ke_human_ = CL/Vd = 4.95/391 = 0.0127 h^−1^. Since local drug transport is highly dependent on muscle blood flow, the clearance of the drug in the human body (CL_trans_) can be converted from the ratio of muscle blood flow between humans and dogs. According to Rowell’s study, the muscle blood flow in dogs is about 300–400 mL 100 g^-1^ min^-1^, while that of the human body is about 73 mL 100 g^-1^ min^-1^ (Rowell, 1988). Since there was no data available for the local blood flow in muscle in dogs, nor in humans, the average blood flow of the muscles of the whole body at rest (taken as 350 mL 100 g^-1^ min^-1^) was used for the calculation of CL_trans_. Based on the ratio of muscle blood flow between humans and dogs, the CL_trans_ of humans was calculated as CL_trans_ = (73/350) × 35 = 7.3 L/h. According to the relationship between the Diffusion Coefficient and the local fluid velocity (Yang et al., 2004), the Diffusion Coefficient in the human body was also adjusted according to the above proportions as D = (73/350) × 4.69 × 10^−12^ = 9.78 × 10^−13^ m^2^/s. Distribution and elimination parameters in human are shown in Table 4.

**TABLE 4:** Physicochemical and pharmacokinetic parameters of paliperidone in human.

Parameters	Symbol	Value
Diffusion Coefficient	D	9.78 × 10^−13^ m^2^/s
Clearance of paliperidone distributed into blood	CL_trans_	7.3 L/h
Elimination rate constant	K_e,human_	0.0127 h^−1^
Volume of distribution	Vd_human_	391 L

**FIGURE 5:**
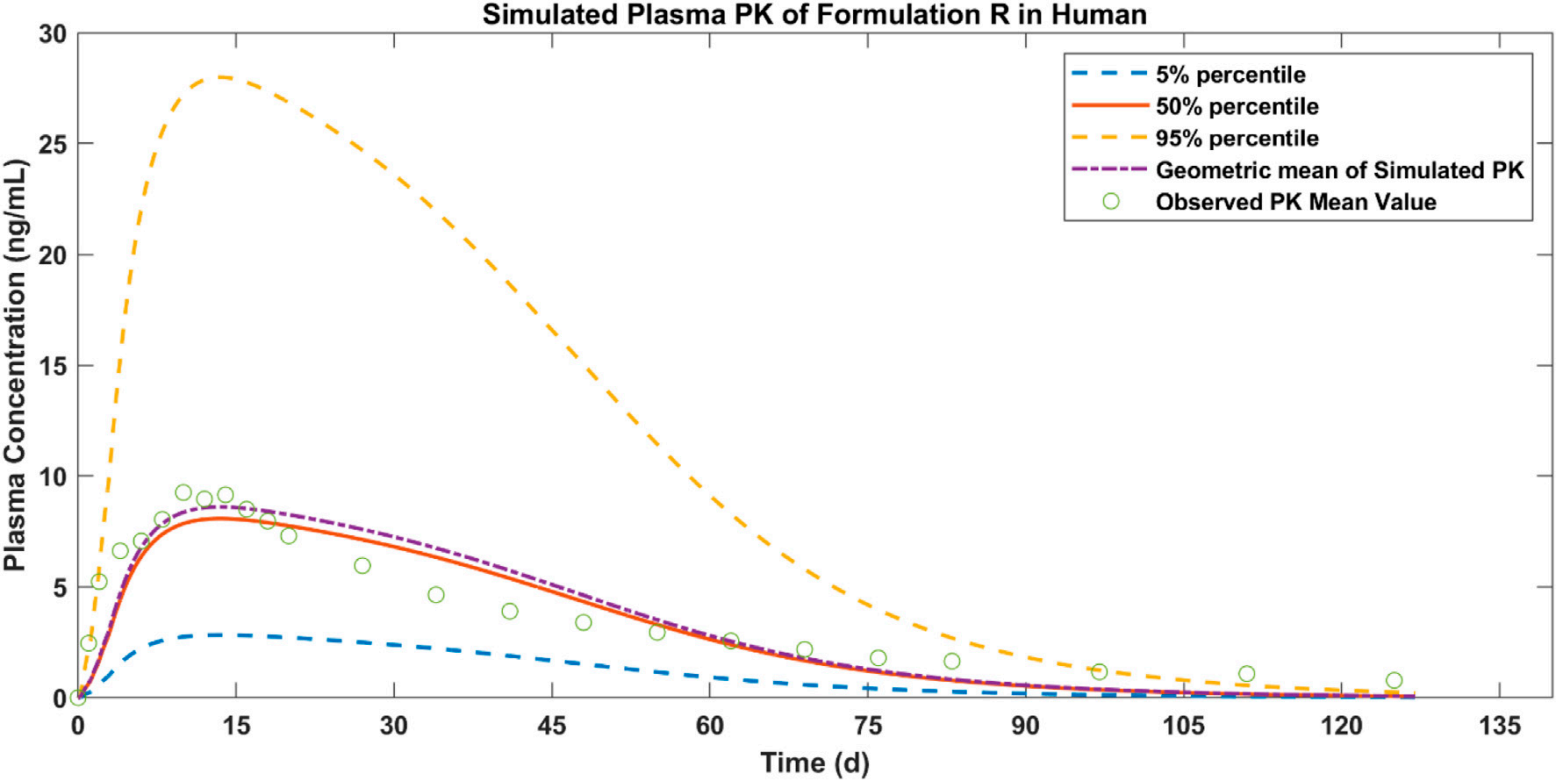
Simulated results of plasma concentration of paliperidone in human (red line) and comparison with observed mean PK values of formulation R (circles).

The plasma concentration of paliperidone in humans after 50 mg injection was simulated and the results are shown in Figure 5. Compared to the observations (circles), during the first 75 days, the simulated plasma concentration of paliperidone (50% percentile, red line) was consistent with the observations in humans. The difference between the simulated results and the observations was less than 2 folds. The simulated paliperidone concentration (50% percentile, red line) dropped to 0.1 ng/mL around 120 days. The AUC_0-t_ and C_max_ values calculated from the concentration curve of the reference sample R are shown in Table 5.

**TABLE 5:** Comparison of simulated and observed results in humans.

Batch	Simulated AUC_0-t_ (ng/mL*hr)	Observed AUC_0-t_ (ng/mL*hr)	Ratio^a^	Simulated C_max_ (ng/mL)	Observed C_max_ (ng/mL)	Ratio^b^
R	10,009	10,062^c^	0.995	8.605	9.257^c^	0.930
T1	8,324	—	—	8.234	—	—
T2	7,220	—	—	7.877	—	—

^a^ Ratio: the ratio of the simulated AUC_0-t_ to observed.

^b^ Ratio: the ratio of the simulated C_max_ to observed.

^c^ Observed data were obtained from the study by Samtani et al., 2009.

### 3.4 Analysis of pharmacokinetic data of test samples T1 and T2 in dogs

Two test samples, T1 and T2, were injected into the musculature of the buttocks of dogs to study the pharmacokinetic behaviour of paliperidone. Based on the pharmacokinetic analysis in the dogs, the volume of distributions (Vd_dog_) of the two samples were fitted and calculated to be 8.5 L, and their bioavailability was 83% and 72%, respectively. These values, along with the volume of distribution and bioavailability (F) of the reference sample, are presented in Table 6. Additionally, the lag time and particle size for each bin of T1 and T2 were derived from their pharmacokinetic analysis, with detailed results provided in Tables 2, 7. Comparing the nanocrystal particle sizes between T1 and T2, each bin presented similar sizes, with similar median diameters (D_50_) of 0.904 μm and 0.827 μm, respectively. For both samples, 90% of the particles (D_90_) were found to have diameters less than 2.3 μm (Table 8). Even though reference samples have D_50_ and D_90_ similar to those of test samples T1 and T2, from Table 7, we can see that after bin 11, the particle size of the reference sample was bigger than those of samples T1 and T2. Details of the D_50_ and D_90_ for test samples and reference samples are listed in Table 8.

**TABLE 6:** Bioavailability and volume of distribution of reference (R) and test (T1 and T2) paliperidone samples in dogs.

Name	Vd_dog_ (L)	Bioavailability F
R	8.5	100%
T1	8.5	83%
T2	8.5	72%

**TABLE 7:** Particle sizes (μm) of reference and test samples in 12 bins.

Batch name	Bin 1	Bin 2	Bin 3	Bin 4	Bin 5	Bin 6	Bin 7	Bin 8	Bin 9	Bin 10	Bin 11	Bin 12
R	0.584	1.24	1.66	2.25	3.03	3.81	4.67	5.68	6.94	8.66	11.4	17.1
T1	0.592	1.18	1.56	2.08	2.75	3.42	4.14	4.98	6.03	7.45	9.65	14.4
T2	0.547	1.10	1.46	1.96	2.60	3.24	3.94	4.76	5.77	7.14	9.29	13.9

**TABLE 8:** Comparison of particle sizes of reference and test samples.

Name	D_10_ (μm)	D_50_ (μm)	D_90_ (μm)
R	0.339	0.853	2.48
T1	0.349	0.904	2.3
T2	0.323	0.827	2.18

**FIGURE 6:**
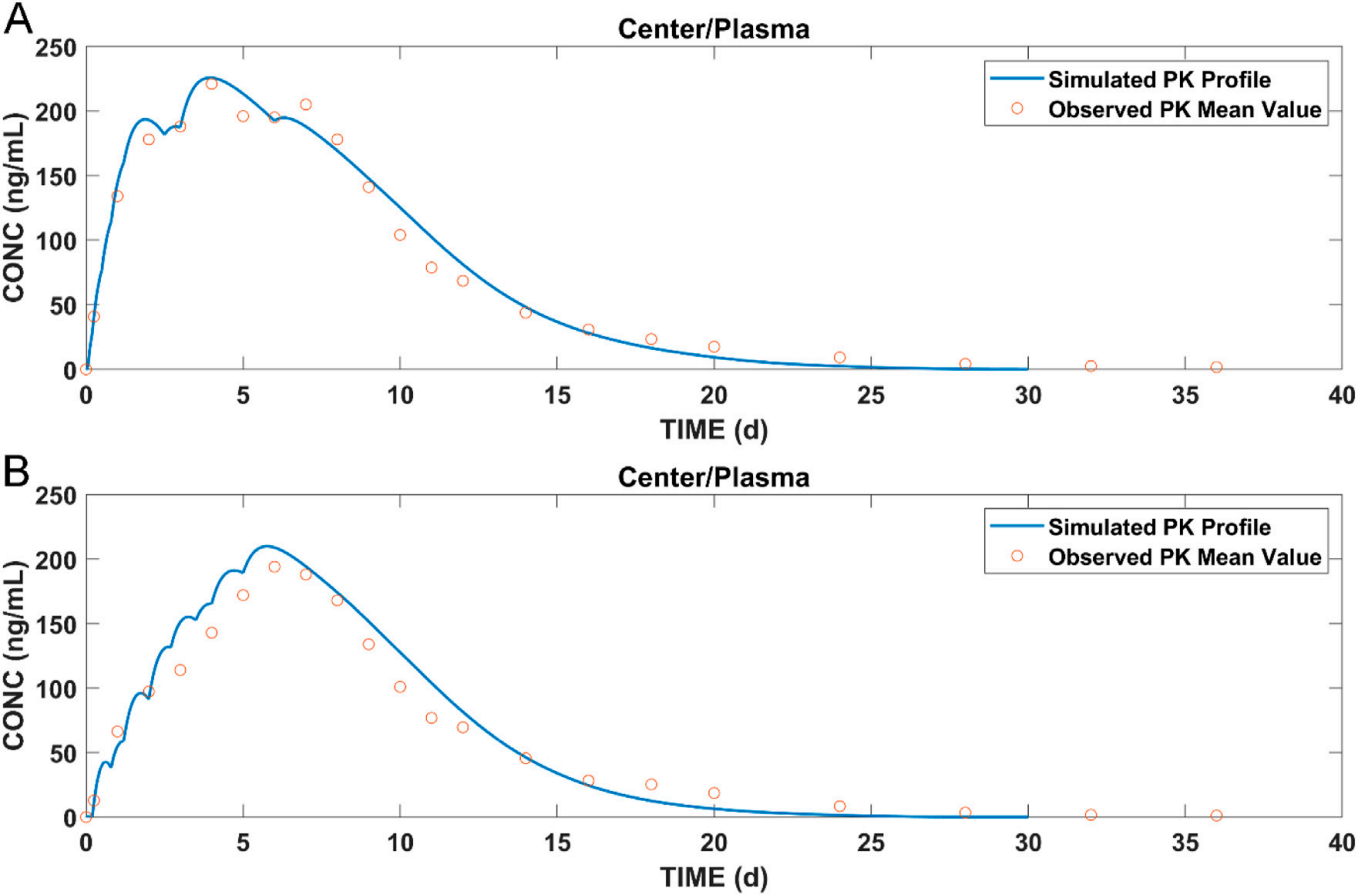
Simulated results of **(A)** plasma concentration of paliperidone for T1; **(B)** plasma concentration of paliperidone for T2; blue lines: simulated results; circles: observed pharmacokinetic data in dogs.

By combining data from pharmacokinetic studies and *in vitro* experiments, the models for T1 and T2 were developed and the simulation results of paliperidone plasma concentrations are shown in Figure 6.

### 3.5 Simulation of the plasma concentrations for T1 and T2

**FIGURE 7:**
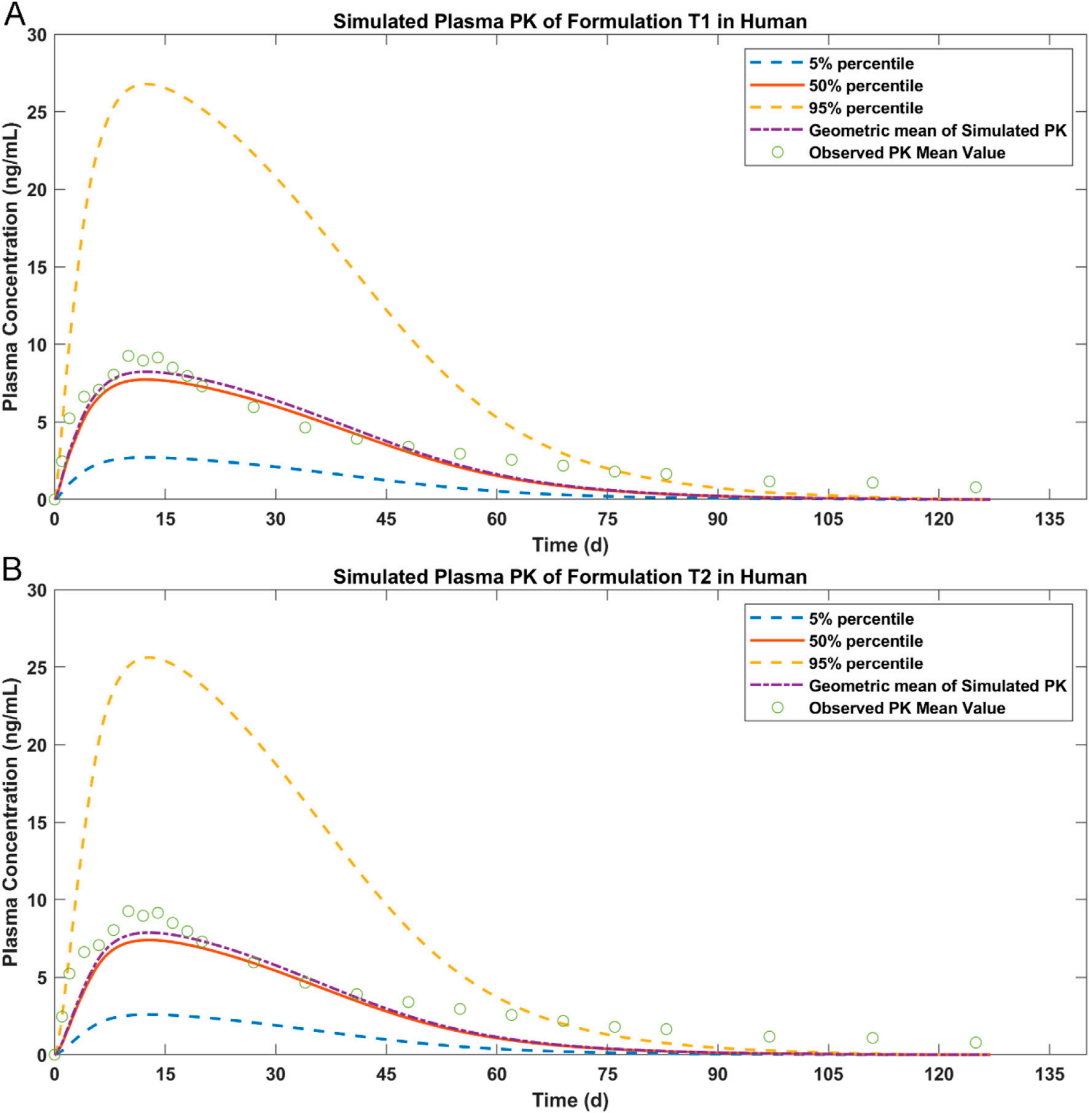
Simulated results of paliperidone in human after IM injection. **(A)** plasma drug concentration for T1; **(B)** plasma drug concentration for T2.

The simulation results of paliperidone after IM injection in humans are shown in Figure 7. Test samples T1 and T2 showed sustained paliperidone release in plasma for approximately 90–100 days (T1: 100 days; T2: 93.8 days). For both samples, the maximum concentration of paliperidone at a dosage of 50 mg has a 50% probability of reaching 7 ng/mL in 10 days. On Day 75, the paliperidone concentration in T1 dropped to 0.6 ng/mL (50% percentile) and in T2 dropped to 0.4 ng/mL (50% percentile). The total release time for T1 or T2 was shorter than the paliperidone reference sample R which sustained release of paliperidone for approximately 120 days (Figure 5).

## 4 Discussion

In contrast to interspecies allometric scaling, which primarily relies on body size and power laws to extrapolate drug kinetics from animals to humans, PBPK modelling simulates drug disposition by accounting for the physiological and biochemical variations across different species. The models incorporate mechanisms of drug absorption, distribution, metabolism, and excretion (ADME). By using data specific to each species, PBPK models can provide more precise predictions when scaling drug behaviours from animals to humans (Chen et al., 2016). In addition, these models support dynamic simulations over time, providing a comprehensive overview of drug concentrations in different tissues. It has been challenging to predict the release of long-acting formulations *in vivo*. Different approaches have been tried to study this topic. In 2015, one research team added a submuscular injection reservoir to the whole-body PBPK model to simulate the absorption of a long-acting formulation in the human body (Rajoli et al., 2015). The model assumed that the drug release had a first-order release constant, which means that the rate at which the drug was released was proportional to the amount of drug remaining in the injection. It was validated against available clinical data and subsequently used to predict the PK of long-acting injectable nanoformulations. However, in other cases, the drug release rate from an extended-release formulation may vary, resulting in complex plasma concentration profiles following release. In 2021, another research group used the convolution approach to solve the problem of prediction of long-acting preparations in case of complex pharmacokinetic profiles (Gomeni and Bressolle-Gomeni, 2021). The researchers used a non-parametric method to predict drug release *in vivo* using this convolution method. The profile of the drug plasma concentration versus time in the human body was directly analysed, and the curve was divided into rectangular segments, arranged sided by side. Between T_i_ and T_i+1_, the release rate of the segment was calculated. T was the time, and i was the i-th segment. The final release rate was obtained based on the comprehensive analysis of the segment release rates. The plasma concentration profile of the long-acting drug in the human body was required before the model was established.

In real-life scenarios, the nanocrystals form a reservoir in the muscle tissue after injection. At this site, nanocrystals begin to dissolve slowly in the muscle’s interstitial fluid. The rate of dissolution is controlled by the size of the nanocrystals and the properties of the drug molecule. After being released, the drug is absorbed into the bloodstream through the capillaries in the muscle. The rate and extent of absorption depend on the drug’s molecular characteristics and the formulation of the nanocrystals (Buxton et al., 2017; Zhang J. et al., 2021). Smaller nanocrystals dissolve more quickly due to their increased surface area-to-volume ratio. With a greater proportion of their surface exposed compared to their volume, these smaller particles provide more area for the solvent to act on, thereby speeding up the dissolution process. This leads to quicker absorption and a more rapid onset of action in the body. When comparing the particle sizes of the samples (T1 and T2) to the reference sample R, T1 and T2 have relatively smaller particle sizes (Table 7) and this explains the faster releases of paliperidone into the plasma.

In this semi-mechanical muscle compartment model, the release of paliperidone *in vivo* was predicted by segmenting the nanocrystals according to their different sizes (bin 1 to bin 12), fitting of the PK study results in dogs to obtain different absorption delays, and then bringing these different absorption delays into the model. As a result, the model can reasonably predict the long-acting nanocrystal injectables in the first 75 days. Increasing the number of bins may lead to a simulated PK curve closer to the observations in dogs, and thus lead to a more accurate simulation of the PK behaviour of the drug when extrapolated to human model. However, adding more bins will decrease computational efficiency, and 12 bins is sufficient to give reasonable simulation results while ensuring computational efficiency. In the present study, an assumption was made in scaling from dogs to humans directly based on blood flow clearance and diffusion coefficients, disregarding physiological differences between humans and dogs. Human muscle-specific blood flow distribution and regulation mechanisms (e.g., slow/fast muscle ratio) are different from those of dogs, and relying only on total blood flow adjustment may lead to biased clearance estimates; also, differences in diffusion-related tissue properties such as fiber structure, cellular interstitial space, and membrane permeability between human muscle and dogs may significantly affect the diffusion efficiency of the drug or metabolite, making extrapolation of the diffusion coefficients subject to the risk of failure. These factors can affect the uncertainty of scaling parameters. Other possible limitations of this model include: 1. Because the release of paliperidone was slow and complex, the limited literature information on distribution and elimination in dogs and the flip-flop phenomenon of IM administration, which makes the fitting of distribution and elimination parameters difficult. Therefore, we assumed that one compartment model was the best choice in the fitting; 2. Information was limited in the literature. Information such as PK data for oral and intravenous paliperidone would further facilitate the fitting of parameters; 3. Because the number of bins was not infinite, the simulated PK curve was close to the observed data only when paliperidone plasma concentration was high. Clinically, exposure to areas of low concentrations may be related to efficacy, and the current model may lead to biased judgments about the efficacy of this subset.

## 5 Conclusion

This article provides a novel approach to modeling and simulating complex and multiphasic drug PK to facilitate the development of long-acting formulations. By segmenting the nanocrystalline particles according to their sizes, the absorption delay of each segment was obtained from the results of the PK study in dogs and introduced into the model for human studies. By using our model, the injectable suspensions of paliperidone were predicted to exhibit a long release of 90–100 days *in vivo*. In the future, we will further improve the model’s predicting power and generalization by training the model on more cases and testing more cases on it, achieving its wider applications.

## Data Availability

The raw data supporting the conclusions of this article will be made available by the authors, without undue reservation.
